# Supplementary material for: A generally conserved response to hypoxia in iPSC-derived cardiomyocytes from humans and chimpanzees
Source: eLife. 2019 Apr 8;8:e42374. doi: 10.7554/eLife.42374 (PMC6538380; doi:10.7554/eLife.42374)
Supplement: Supplementary file 1. [file elife-42374-supp1.docx]

**Table S1: Processing information for each sample.** Each sample and its sex (F-female; M-male), CO_2_ level during the course of the experiment (K-known: 5% CO_2_; U-unknown), the presence of episomal reprogramming vector in the iPSC line, oxygen perturbation experiment batch, RNA extraction batch, sequencing library preparation batch, sequencing pool, and cellular assay plates are shown. ‘R’ denotes replicate experiments. All four conditions associated with a sample were processed together.

**Table S2: Summary of the data generated in this study.** RIN scores, conservative and lenient estimates of cardiomyocyte purity, oxygen level, and numbers of sequencing reads are shown.

**Table S3: Numbers of differentially expressed genes, and genes within expression clusters. A)** The numbers of differentially expressed genes between contrast pairs obtained from the limma analysis are shown. The numbers for the full set of samples (7 chimpanzees (C) and 8 humans (H)), as well as a subset of the data (5 chimpanzees and 5 humans) are shown. **B)** The number of genes in the four gene expression clusters determined by Cormotif.
